# Prevalence of anxiety and depression in patients with acute coronary syndrome: systematic review and meta-analysis

**DOI:** 10.11604/pamj.2023.46.91.41792

**Published:** 2023-11-26

**Authors:** Percy Junior Castro Mejía, Pietro Dondero Cassano, Percy Díaz Morón, Mónica Díaz Reátegui, Karem Menacho Navarrete, Pedro Córdova-Mendoza

**Affiliations:** 1 Universidad Cesar Vallejo, Chiclayo, Perú,; 2 Facultad de Ingeniería y Negocios, Universidad Norbert Wiener, Lima, Perú,; 3 Universidad Señor de Sipán, Chiclayo, Perú,; 4 Escuela de Medicina, Universidad César Vallejo, Lima, Perú,; 5 Universidad Nacional San Luis Gonzaga, Ica, Perú

**Keywords:** Anxiety, depression, acute coronary syndrome, systematic review

## Abstract

Depression and anxiety are common in patients experiencing acute coronary syndrome (ACS), occurring at significantly elevated rates. Together, these depressive symptoms and anxiety have a substantial negative impact on individuals with ACS. Therefore, the aim of the present study was to determine the prevalence of anxiety and depression among patients with ACS. A systematic review and meta-analysis of cross-sectional studies were carried out. A comprehensive search of five databases (PubMed, Scopus, Embase, Web of Science, and ScienceDirect) was performed until August 2, 2023. The quality of the included studies was assessed using the Joanna Briggs Institute statistical meta-analysis review instrument. The collected data were entered into a Microsoft Excel spreadsheet and analyzed with the R program version 4.2.3. A total of 3103 articles were evaluated, and, after the evaluation process, eight studies were included, for a total sample of 1642 participants. The pooled prevalence of mild depression was 14% (95% CI: 06%-23%; I^2^= 95%), moderate was 12% (95% CI: 06%-19%; I^2^= 92%), and high/severe was 15% (95% CI: 05%-30%; I^2^= 97%). The joint prevalence of mild anxiety was 38% (95% CI: 12%-68%; I^2^= 98%), moderate anxiety was 17% (95% CI: 08%-29%; I^2^= 89%), and high/severe anxiety was 10% (95% CI: 01%-25%; I^2^=95%). Therefore, it is concluded that there is a significant prevalence of anxiety and depressive symptoms in patients with ACS. However, more research focused on this area is required to obtain more robust and substantial evidence.

## Introduction

Acute coronary syndrome (ACS) is a general term used to describe any condition that decreases blood flow to the heart muscle, leading to the appearance of signs and symptoms characteristic of acute myocardial ischemia [1]. These manifestations include unstable angina, non-ST-segment elevation myocardial infarction, and ST-segment elevation myocardial infarction. These disorders can progress to severe complications, including heart failure or even death [2]. Currently, ACS represents the primary causal factor in cardiovascular disease-related deaths worldwide [3], impacting a total of 7.4 million deaths worldwide, representing 21-22% of all deaths in Europe and 6-10% of deaths in sub-Saharan Africa [4]. According to the World Health Organization (WHO) in its 2023 report, cardiovascular diseases are responsible for approximately 17.9 million deaths worldwide. Patients suffering from these diseases experience a marked decrease in their quality of life, which can lead to the development of psychiatric disorders such as anxiety and depression [5]. According to research, it has been observed that people suffering from cardiovascular disease present levels of anxiety and depression, with a prevalence ranging from 20% to 45% [6]. These values are notably higher compared to the incidence rates in the general population, being 3 to 4 times higher. The European Society of Cardiology and a Scientific Statement of the American Heart Association have jointly recognized the growing impact of psychosocial factors, such as depression and anxiety, on the development and progression of cardiovascular disease [7-9]. Anxiety symptoms are common among patients with ACS. The prevalence of anxiety in these patients reaches about 56.5% [3]. According to the findings of one study, it has been observed that more than 33% of individuals with ACS experience significantly elevated levels of anxiety [10]. Anxiety influences the functioning of body organs, decreases quality of life, and aggravates cardiovascular health status in patients who have experienced myocardial infarction [11,12]. The presence of depressive symptoms significantly increases the risk of mortality in patients with coronary heart disease. In fact, patients who experience depressive symptoms are twice as likely to die within 2 years of their initial evaluation compared with those who do not have depressive symptoms [13]. About 20% of ACS sufferers experience a major depressive disorder, and approximately two out of three patients who undergo ACS still show noticeable depressive symptoms several months after the incident [14,15]. Understanding anxiety and depression in patients with ACS is critical because of their profound impact on the recovery process and long-term outcomes [16]. Anxiety and depression not only worsen patients' quality of life but are also linked to a significant increase in cardiovascular morbidity and mortality [17]. The aim of the investigation was to establish the prevalence of anxiety and depression among patients with acute coronary syndrome, using a categorization of anxiety and depression levels ranging from mild to moderate to high. Levels of anxiety and depression, ranging from mild to moderate to high, can influence treatment compliance and adherence to lifestyle changes. In addition, anxiety and depression can trigger negative physiological responses, such as increased oxidative stress and inflammation, which can aggravate the progression of heart disease [18]. Therefore, careful assessment of anxiety and depression levels in ACS patients and categorizing these levels appropriately is essential to implementing early and personalized interventions that address both cardiac problems and mental conditions, thereby improving quality of life and reducing the risk of future complications [19].

## Methods

**Protocol and registration:** this systematic review and meta-analysis has been registered in the Prospective International Registry of Systematic Reviews (PROSPERO) database (CRD42023456093). For manuscript preparation, the Preferred Reporting Items for Systematic Reviews and Meta-analyses (PRISMA) guidelines have been followed.

**Eligibility criteria:** the analysis included published articles that met the following criteria: i) cross-sectional studies reporting the prevalence of depression or anxiety at mild, moderate, and severe levels; ii) participants with acute coronary syndrome diagnosed on the basis of clinical manifestations, electrocardiography, and cardiac biomarkers. On the other hand, studies with designs other than cross-sectional and those that reported the prevalence of depression and anxiety without a standardized classification into mild, moderate, and severe categories were excluded. In addition, patients without a definitive diagnosis of ACS or with any psychiatric disorder were excluded.

**Information sources and search strategy:** the search strategy focused on evaluating the presence of depression and anxiety in patients with acute coronary syndrome, categorizing them into low, moderate, and severe levels. A systematic literature search was conducted in the PubMed, Scopus, Embase, Web of Science, and ScienceDirect databases until August 2^nd^. Without applying any time or language restrictions, we used MeSH terms for each of the databases, such as “depression”, “anxiety”, and “acute coronary syndrome”. The bibliographic search and article selection process were independently carried out by two authors, and there were no significant disagreements regarding the inclusion of studies (Annex 1).

**Study selection:** the articles obtained as a result of the search in the five databases were transferred to Rayyan for management and storage. First, duplicate articles were removed, and then they were evaluated by reviewing their titles and abstracts, following pre-established criteria. Subsequently, a full-text review was conducted for those that met the established inclusion criteria. Any discrepancies were resolved through discussions and consultations with a researcher.

**Main results of the study:** depression and anxiety according to the low, moderate, and severe levels of patients with acute coronary syndrome

**Quality assessment:** the “JBI-MAStARI” method was used to assess the quality of the included cross-sectional studies. These studies were classified into three categories based on their quality scores: high (score ≥ 7 points), moderate (score of 4 to 6 points), or low (score < 4 points) (Annex 2) [20].

**Data collection process and data items:** we proceeded to extract and tabulate a wide variety of features from each retrieved article into an Excel spreadsheet. These features included the name of the main author, the year of publication, the country where the study was conducted, the sample used, as well as the classification of depression (mild, moderate, severe/high) and anxiety (mild, moderate, severe/high). Additionally, additional data such as study subjects' age and gender (male and female) were recorded. Whenever it was necessary to clarify the results or conduct additional analyses, efforts were made to contact the authors of the articles. However, varying levels of success were observed in this process, depending on the availability and responsiveness of the contacted authors. To ensure the accuracy of the data extracted from the included articles, three independent researchers reviewed and verified the data. Any discrepancies that arose were resolved through discussion with a fourth researcher.

**Data analysis:** the R software, version 4.2.3, was used to perform data analysis of the included articles. The results were presented through tables and graphs. The pooled prevalence of depression and anxiety in patients with acute coronary syndrome was determined using the random-effects model with inverse variance weighting. To assess heterogeneity among the studies, the Cochrane Q statistic was employed, and its quantification was done using the I^2^index. To examine potential publication bias, funnel plots were used, and the Egger's regression test was applied. The presence of possible publication bias was considered when the p-value was less than 0.05 [20].

## Results

**Figure 1:**
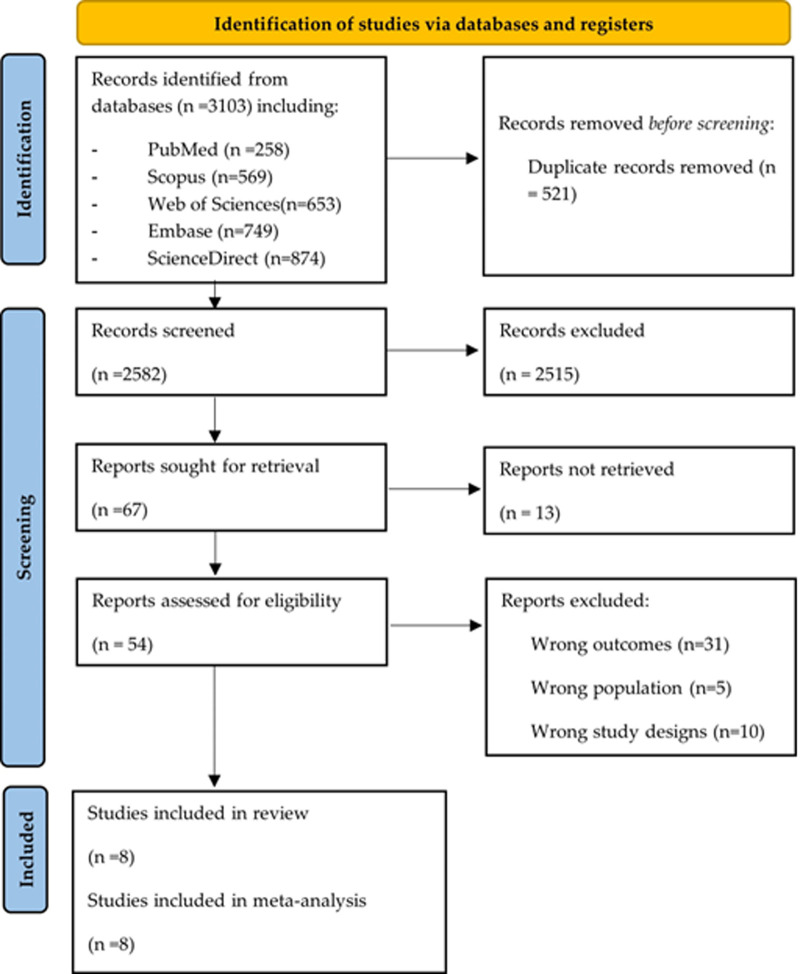
flow chart of the study selection process

**Study selection:** the exhaustive search process yielded a total of 3103 articles, among which 521 duplicates were identified. Subsequently, the selection of titles and abstracts was performed for these 2582 articles, and after a careful evaluation of their eligibility, a full-text examination was conducted on 54 articles. After this analysis, 46 articles that did not meet the required criteria were excluded. Finally, a group of 8 eligible studies was identified and included for systematic review and meta-analysis [21-28]. The entire process of article review and selection is depicted in the PRISMA flow diagram (Figure 1).

**Characteristics of the included studies:** eight cross-sectional studies published between 2005 and 2022 were included, with a total sample of 1642 participants. These studies were conducted in 7 countries: Peru, Malaysia, Pakistan, United States, Serbia, Australia, and Denmark. Seven studies were evaluated, reporting the presence of depression in their study patients, and classifying it into mild, moderate, and severe levels. Additionally, five studies reported on anxiety in their participants, also classifying it into mild, moderate, and severe levels. Of the total sample, 66.44% were male and 33.56% were female (Table 1) [21-28].

**Table 1:** characteristics of included studies reporting anxiety and depression in patients with acute coronary syndrome

Authors	Year	Study design	Country	Sample size	Depression (n, %)			Anxiety (n, %)			Study subjects	Age (Years)	Sex	
					Mild	Moderate	High/severate	Mild	Moderate	High/severate			M	F
Furlong-Millones MR *et al*.	2022	Cross-sectional	Peru	85	29 (34.1%)	17 (20.0%)	21 (24.7%)	67 (78.8%)	11 (12.9%)	7 (8.3%)	General population	<60: 37 (43.5%) ≥60: 48 (56.5%)	65	20
Leong LK *et al*.	2021	Cross-sectional	Malaysia	95	23 (24.2%)	13 (13.7%)	31 (32.6%)	NR	NR	NR	General population	<60: 42 (44.2%) ≥60: 53 (55.8%)	69	26
Hadi N *et al*	2020	Cross-sectional	Pakistan	110	15 (13.6%)	20 (18%)	56 (50.9%)	37 (33.6%)	36 (32.7%)	37 (33.6%)	General population	NR	75	35
Mujtaba SF *et al*.	2020	Cross-sectional	Pakistan	153	11 (7.2%)	6 (3.9%)	1 (0.7%)	26 (17%)	10 (6.5%)	2 (1.3%)	General population	Mean: 52.15	118	35
Yammine L *et al*.	2014	Cross-sectional	United States	153	22 (14.4%)	27 (17.6%)	15 (9.8%)	NR	NR	NR	General population	Mean: 45.9	85	68
Ciric-Zdravkovic SV *et al*.	2014	Cross-sectional	Serbia	30	NR	NR	NR	21 (70%)	9 (30%)	0 (0%)	General population	Mean: 61.75	19	11
DiGiacomo M *et al*.	2007	Cross-sectional	Australia	117	12 (10.3%)	15 (12.8%)	12 (10.3%)	6 (5.3%)	15 (13.2%)	25 (21.1%)	General population	Range: 55-70	0	117
Sørensen C *et al*.	2005	Cross-sectional	Denmark	899	25 (2.8%)	34 (3.8%)	31 (3.4%)	NR	NR	NR	General population	NR	660	239

NR: not reported

**Quality of the included studies and publication bias:** all studies presented high methodological quality (Annex 2) [21-28].

**Prevalence of depression:** the combined prevalence of mild depression in patients with acute coronary syndrome was 14% (95% CI: 06%-23%; 1612 participants; 7 studies; I^2^= 95%) (Annex 3) [21-25,27,28]. Additionally, the combined prevalence of moderate depression in these patients was 12% (95% CI: 06%-19%; 1612 participants; 7 studies; I^2^= 92%) (Annex 4) [21-25,27,28], while the combined prevalence of high/severe depression was 15% (95% CI: 05%-30%; 1612 participants; 7 studies; I^2^= 97%) (Annex 5) [21-25,27,28].

**Prevalence of anxiety:** it was observed that the overall prevalence of anxiety in patients with acute coronary syndrome varied at different levels. In the case of mild anxiety, a rate of 38% was recorded (95% CI: 12%-68%; 495 participants; 5 studies; I^2^=98%) (Annex 6) [21,23,24,26,27]. For moderate anxiety, the overall prevalence was 17% (95% CI: 08%-29%; 495 participants; 5 studies; I^2^= 89%) (Annex 7) [21,23,24,26,27], while for high/severe anxiety, it was 10% (95% CI: 01%-25%; 495 participants; 5 studies; I^2^= 95%) [21,23,24,26,27].

## Discussion

Acute coronary syndrome is a high-risk disease of sudden onset with significant severity, rapid evolution, and a high rate of mortality and disability [29]. Anxiety disorders and depression represent significant risk factors for cardiovascular mortality in patients with coronary artery disease (CAD). Acute coronary syndrome is listed as the primary clinical manifestation in the setting of evolving CAD [6]. A scoping review has pointed out that the onset of ACS increases the likelihood of experiencing depressive or anxiety disorders in the first one to two years after the acute event. In addition, both depression and anxiety are associated with an increased risk of additional episodes of acute coronary problems [19]. This is why the present systematic review and meta-analysis aim to determine the prevalence of anxiety and depression in patients with ACS. Taken together, these results suggest that depression is relatively common among patients who have suffered from ACS. Mental health is an important aspect to consider in the treatment and care of these patients, as depression can have negative effects on recovery and overall quality of life. It is important for health care professionals to be aware of these findings and consider the assessment and management of the mental health of patients who have experienced an ACS. The results of the study on the combined prevalence of anxiety revealed 38% for mild, 17% for moderate, and 10% for high or severe anxiety in patients with ACS. Tran H *et al*. reported in their multicenter study in the United States that in patients with ACS, 10.4% had a documented diagnosis of anxiety and 18.8% had moderate or severe symptoms of anxiety [30]. A meta-analysis study of 39,338 patients with ACS reported that the presence of anxiety was independently associated with an increased risk of mortality and adverse cardiovascular events [31]. Another meta-analysis reported an overall estimate of the prevalence of anxiety of 32.9% [32]. Anxiety is a widely recognized autonomic risk factor in relation to adverse cardiovascular events and mortality in ACS. However, how stress-triggered anxiety behavior impacts the metabolic profile of blood plasma and how it contributes to the deterioration of coronary artery disease has not been precisely elucidated [33]. The results of the present study established that the combined prevalence of depression in patients with acute coronary syndrome was 14% for the mild form, 12% for the moderate form, and 15% for the high or severe form. Yuan J *et al*., in their cross-sectional study of 782 Chinese patients with ACS, reported a prevalence of major depression of 15.6% [34]. A meta-analysis reported an overall estimate of the prevalence of depression of 31.3% [32]. Several investigations have consistently shown that the prevalence rate of depression in ACS patients is around 20%, and depressive symptoms, such as distress or mild depression, show even higher rates [35]. Taken together, these results suggest that anxiety is common among patients with ACS and varies in severity. This highlights the importance of considering anxiety as a factor in the medical care of these patients, as it may have implications for both their psychological well-being and physical recovery. Healthcare professionals can use this information to identify patients who may need additional support in terms of anxiety management and improve the quality of care provided to this population. These findings are of great importance as they provide key guidance for the formulation of public health strategies aimed at reducing mental health problems among patients with ACS. A connection has been established between depression and anxiety, with increased risks associated with quality of life as well as adverse outcomes and higher medical expenditures in those individuals suffering from ACS. These findings underscore the need to comprehensively address both medical and emotional aspects in the management of patients with ACS in order to improve clinical outcomes and reduce the economic burden on the health care system [36].

Furthermore, detailed categorization of anxiety and depression levels among patients with acute coronary syndrome is essential for comprehensive, patient-centered medical care. Mild, moderate, and high levels of anxiety and depression require different approaches in terms of therapeutic and emotional support interventions. Patients with mild levels may benefit from psychoeducational interventions and cognitive-behavioral therapy, while those with moderate and high levels may need more intensive interventions, such as pharmacological therapy or intensive cognitive-behavioral therapy [37]. In addition, accurately identifying these levels also helps healthcare professionals recognize additional risk factors and customize the treatment and rehabilitation plan. By addressing both cardiac and mental conditions with a holistic and tailored approach to anxiety and depression levels, the quality of care is significantly improved, and the chances of a successful recovery for patients with acute coronary syndrome are maximized [38-40]. This study has some limitations. We found a limited number of studies assessing the prevalence of anxiety and depression among ACS patients, employing a categorization of anxiety and depression levels ranging from mild to moderate to high. Therefore, due to the limited number of eight original papers, it was not feasible to perform a publication bias analysis. In this investigation, a random-effects model was employed in order to address the considerable variation that resulted in heterogeneity across studies. Among the strengths of this study is its systematic nature. This review represents a milestone as the first analysis aimed at evaluating two highly relevant mental health pathologies, specifically anxiety and depression, in patients with ACS. The study was developed following a rigorous methodology based on PRISMA guidelines, and a thorough quality assessment of the studies incorporated in the analysis was carried out. Finally, it is important to conduct comprehensive research to thoroughly understand the prevalence of anxiety and depression in patients who have experienced ACS. These psychological conditions can significantly influence the physical and emotional recovery of patients who have suffered an acute cardiovascular event. Understanding the interaction between ACS and anxiety and depressive disorders is crucial to developing effective and personalized interventions that address both the cardiac and mental needs of these patients. In addition, future research should explore specific psychosocial risk factors that might contribute to the development of anxiety and depression in this population, thus allowing for a more preventive and targeted approach to the underlying causes of these conditions. In addition, it is essential to conduct longitudinal studies assessing the long-term relationship between ACS and anxiety and depressive disorders. These investigations could help identify patterns and trends over time, allowing early and ongoing intervention to improve patients' quality of life. In addition, demographic factors, such as age and gender, should be considered to better understand how these variables may affect the prevalence and management of anxiety and depression in patients with ACS. Future research in this field will not only improve our understanding of the intersection between cardiac and mental health but will also provide a solid foundation for the development of more effective, patient-centered treatment strategies.

## Conclusion

The present study determined a joint prevalence of mild anxiety (38%), moderate anxiety (17%), and high/severe anxiety (10%) in ACS patients. In addition, a joint prevalence of mild depression (14%), moderate depression (12%), and high/severe depression (15%) in ACS patients was determined.

### What is known about this topic

- *Cardiovascular diseases present levels of anxiety and depression, with a prevalence ranging from 20% to 45%;*
- *Acute coronary syndrome (ACS) represents the primary causal factor in cardiovascular disease-related deaths worldwide;*
- *Depression and anxiety are common among patients facing ACS, having a significantly adverse impact on the quality of life of those who suffer from it*.

### What this study adds

- *The prevalence of depression experienced by patients with ACS was presented at different levels of mild, moderate, and high/severe, with respective percentages of 14%, 12%, and 15%;*
- *The prevalence of depression experienced by patients with ACS was presented at different levels of mild, moderate, and high/severe, with respective percentages of 38%, 17%, and 10%*.

## Annexes

**Annex 1**: the adjusted search terms as per searched electronic databases (PDF - 78KB)

**Annex 2**: quality of the included studies (PDF - 140KB)

**Annex 3**: forest graph showing the prevalence of mild depression in patients with acute coronary syndrome (PDF - 93KB)

**Annex 4**: forest graph showing the prevalence of moderate depression in patients with acute coronary syndrome (PDF - 99KB)

**Annex 5**: forest graph showing the prevalence of high/severe depression in patients with acute coronary syndrome (PDF - 105KB)

**Annex 6**: forest graph showing the prevalence of mild anxiety in patients with acute coronary syndrome (PDF - 98KB)

**Annex 7**: forest graph showing the prevalence of moderate anxiety in patients with acute coronary syndrome (PDF - 92KB)
